# Multiple drug hypersensitivity to beta lactam and non beta lactam antibiotics, inhibitors of conversion enzyme, thiopental and COX2 inhibitors on a hyperthyroid male patient - case report

**DOI:** 10.1186/2045-7022-4-S3-P137

**Published:** 2014-07-18

**Authors:** Celina Stafie

**Affiliations:** 1 University of Medicine GR.T.POPA Iasi, Allergology and Immunology Department, Romania

## Abstract

The multiple drug hypersensitivity is an rare syndrome, severe as presentation, evolution and therapeutic approach as well as for the patient, as for the allergist. It includes a panoramic approach of the clinical already assessed reactions (severe general urticaria, laryngeal edema, Quincke edema, anaphylaxis).

## Introduction

A case report of an 50 years old patient which started 14 years ago , with an severe onset - glotic edema with anaphylaxis, after a second intake of Rocephin (Sodium Ceftriaxone) and also due to an emotional distress and associated with a malfunction of the thyroid status. He develops within a 3 year period of time a multiple drug hypersensitivity syndrome to beta lactam and non beta lactam antibiotics, inhibitors of converting enzyme, thiopental and COX2 inhibitors. Moreover, he is developping allergic cross reactivity to food or beverages that includes in their components antigenic fractions linked to allergens to which the patient is sensitised. Patient's history includes recurrent episodes of severe general urticaria due to Ceftriaxone and laryngeal edema due to Rofecoxibe, as well as expiratory dyspnoea to non steroidal inflammatory drugs, such as ibuprofen, diclofenac and also to an anaestetic - thiopental. The thyroid status and pshycological type of reaction- as strong choleric, large catecholamines amount releaser recommends him for a detailed test for Ig E, non Ig E, mast cell degranulation test or lymphoblastic transformation test. It is quite improbable that an overview for all drugs for specific Ig E could be performed in terms of financial expenditure.

## Conclusion

The main focus for this patient is to establish a list of "secure" drugs, from each main category of potentially needed - like hypertension or ischemic heart, local anesthesia, intercurrent infections and even gastric ulcer- for the patient has a very high score of BDT for esomeprazol as well. Tests performed for BDT showed a number of more then 15 drugs with a high limit over then 200 (maximum admitted) and another 10 drugs with an interval between 100-150, meaning with high risk to increase also if not avoided.

